# Antibiotic Resistance in *Streptococcus pneumoniae* after Azithromycin Distribution for Trachoma

**DOI:** 10.1155/2015/917370

**Published:** 2015-10-18

**Authors:** Derek K-H. Ho, Christian Sawicki, Nicholas Grassly

**Affiliations:** ^1^Department of Ophthalmology, William Harvey Hospital, Kennington Road, Willesborough, Ashford, Kent TN24 0LZ, UK; ^2^Department of Anesthesia, University of Toronto, 123 Edward Street, Toronto, ON, Canada M5G 1E2; ^3^School of Public Health, Imperial College London, Norfolk Place, London W2 1PG, UK

## Abstract

Trachoma is caused by *Chlamydia trachomatis* and is a leading cause of blindness worldwide. Mass distribution of azithromycin (AZM) is part of the strategy for the global elimination of blinding trachoma by 2020. Although resistance to AZM in *C. trachomatis* has not been reported, there have been concerns about resistance in other organisms when AZM is administered in community settings. We identified studies that measured pneumococcal prevalence and resistance to AZM following mass AZM provision reported up to 2013 in Medline and Web of Science databases. Potential sources of bias were assessed using the Cochrane Risk of Bias Tool. A total of 45 records were screened, of which 8 met the inclusion criteria. We identified two distinct trends of resistance prevalence, which are dependent on frequency of AZM provision and baseline prevalence of resistance. We also demonstrated strong correlation between the prevalence of resistance at baseline and at 2-3 months (*r* = 0.759). Although resistance to AZM in *C. trachomatis* has not been reported, resistance to this commonly used macrolide antibiotic in other diseases could compromise treatment. This should be considered when planning long-term trachoma control strategies.

## 1. Introduction

Trachoma, caused by ocular infection with* Chlamydia trachomatis*, is the leading contagious cause of blindness globally. Estimations by Mariotti et al. in 2008 suggested there were 40.6 million people worldwide suffering from active trachoma, leading to trichiasis in 8.2 million [1]. Trachoma is responsible for visual impairment in 2.2 million and clinical blindness in 1.2 million individuals [2].

Trachoma, a Neglected Tropical Disease (NTD), spreads efficiently within households and in areas with poor sanitation and hygiene [3]. The* Musca sorbens* fly, which breeds on human faeces, may also act as a vector for transmission. The disease is highly correlated with poverty, lack of clean water for washing, and limited access to healthcare. Trachoma infections occur mainly in children aged 1 to 5, who act as reservoir for the bacterium. In older age groups, the development of immunity limits infection, although reduced exposure as a result of behavioural change also contributes to the declining prevalence of infection with age [4].

Repeated ocular infections by* Chlamydia trachomatis* inflame the eyelids, leading to scarring of the conjunctival lining of the upper eyelid. When the lid margin is distorted, eyelashes turn inwards touching the eye surface, termed entropion trichiasis. Unresolved repeated abrasions of the cornea contribute to disabling pain, corneal opacification, and ultimately the loss of vision [1, 5].

In 1997, WHO established the Alliance for Global Elimination of Trachoma by the year 2020 (GET 2020), a partnership with the aim to eliminate blindness caused by trachoma [6]. The GET 2020 alliance recommends interventions for trachoma control, known by the acronym SAFE, which stands for surgery for trichiasis, antibiotics, facial cleanliness, and environmental improvement, including clean water and latrines.

The aim of antibiotic use is not only to treat the affected patients, but also to limit disease transmission to others. It was estimated that the ultimate intervention goals would require antibiotic treatment for some 340 million people and trichiasis surgeries for 8.2 million [1, 5].

There are concerns about the possibility of antibiotic resistance, especially among bacterial pathogens such as* Streptococcus pneumoniae*, which could potentially undermine the SAFE strategy. Invasive pneumococcal diseases associated with* Streptococcus pneumoniae* are a major cause of morbidity and mortality worldwide, resulting in around half a million deaths in children under 5 in 2008, mostly in developing countries [7]. Invasive pneumococcal diseases include pneumonia, meningitis, and bacteraemia. Azithromycin is commonly used for the treatment of community-acquired pneumonia in adults and clinical cure is compromised in patients infected with resistant strains [8].

Resistance to azithromycin (AZM) has not been documented in isolates of* Chlamydia trachomatis* [9] following mass treatment in trachoma control programmes. However, correlation between macrolide use and resistance in* Streptococcus pneumoniae* has been well documented [10, 11]. For example, a randomized controlled trial by Malhotra-Kumar et al. showed a causal effect between AZM use in individuals and resistance in streptococci, which remained significant until 6 months after treatment [11].

Several studies have been conducted to examine the possibility of antibiotic resistance in* Streptococcus pneumoniae* after mass trachoma treatment with oral AZM. Here, we attempt to summarize their findings and quantify the impact of AZM. This systematic review considers studies that involved participants of any age in trachoma-affected regions, who received oral azithromycin as part of trachoma eradication program, where outcome measures included* Streptococcus pneumoniae* presence and antibiotic resistance detected from nasopharyngeal swabs of participants.

## 2. Methods

Searches were performed by Derek K-H. Ho and Christian Sawicki on electronic databases Medline and Web of Science up to 23rd of January 2014. Search terms included azithromycin, Zithromax©, resistance, resistant, and trachoma. The search string was “(azithromycin or zithromax) AND resistan^*∗*^ AND trachoma.” No limits were applied for language or year. Cochrane and DARE (Database of Abstracts of Reviews of Effectiveness) databases were also consulted [12, 13].

### 2.1. Eligibility Criteria

#### 2.1.1. Inclusion Criteria

Studies of community-wide trachoma treatment with azithromycin that measured the prevalence of* Streptococcus pneumoniae* carriage and AZM sensitivity based on nasopharyngeal swabs were included in the analysis. Selection criteria for swabbed individuals should be clearly outlined. Resistance status was based on minimum inhibitory concentration values as per test kit instructions. Any year, language, or length of follow-up were considered. Participants who received other drugs such as tetracycline ointment or took azithromycin in forms other than oral were not included in the analysis.

#### 2.1.2. Outcome Measures

Primary outcome measure is the prevalence of antibiotic resistance to azithromycin in* Streptococcus pneumoniae* isolated from nasopharyngeal swabs of study participants before and after community-wide administrations.

Secondary outcome measure is the prevalence of* Streptococcus pneumoniae* carriage in nasopharyngeal samples from participants.

#### 2.1.3. Exclusion Criteria

The following studies were excluded: reports of antibiotic resistance in species other than* S. pneumoniae*; use of azithromycin other than for treatment and prevention of trachoma; mathematical modeling; surveillance reports; review articles; case reports or series with a study size of less than 50; studies without a consistent laboratory protocol for resistance testing; studies without sufficient information on the number of samples tested.

Eligibility assessment was performed by Derek K-H. Ho and Christian Sawicki independently, the results of which were then checked by Nicholas Grassly.

### 2.2. Data Extraction Process

Derek K-H. Ho extracted the data from the studies that met the eligibility criteria, and then recorded the primary and secondary outcome measures as well as the participant numbers and demography, geographic areas, azithromycin regimes, and its background usage in the regions. The data was then examined by Nicholas Grassly. Derek K-H. Ho also assessed the included studies for risk of bias using the Cochrane Risk of Bias Tool [14].

### 2.3. Statistical Analysis

Odds ratios (OR) were calculated and pooled across studies using the Mantel-Haenszel random effects model implemented in Review Manager 5.2. Results were summarized in forest plots and heterogeneity across studies assessed using the *I* ^2^ statistic [15]. Exact 95% confidence intervals on proportions were calculated using the Clopper-Pearson method.

For the purpose of analysis, we defined resistance to azithromycin as reported in the studies.

## 3. Results

**Figure 1:**
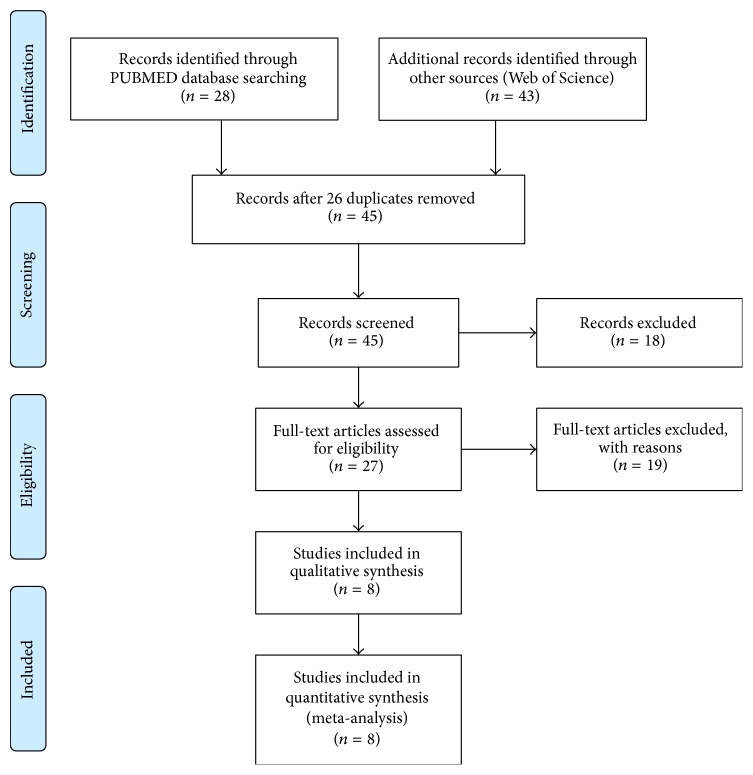
PRISMA flow diagram.

Searches on Medline and Web of Science provided 28 and 43 results, respectively, giving a total of 71 citations. After adjusting for duplicates, 45 remained. Of these, 18 studies were discarded as they did not fit the inclusion criteria, having reviewed their titles and abstracts. The full text of the remaining 27 citations was retrieved and examined in detail, and 19 of them were rejected by the exclusion criteria. A total of 8 remaining studies were identified for inclusion in the systematic review [16–23]. All 8 studies were published in English. No unpublished data were sought. The selection process is depicted as the PRISMA flow diagram (Figure 1).

### 3.1. Characteristics of Included Studies

#### 3.1.1. Participants

The included studies involved around 10000 participants in total. 6 were community-based studies and 2 were individual-based studies (Table 1). One study [23] recruited children under 5, three studies [17, 19, 20] recruited 1- to 10-year-olds, two studies [18, 21] recruited all nonpregnant residents over 1 year old, and two studies [16, 19] recruited children with trachoma and their household contacts. In four studies [18, 20–22], participants who were ineligible, including pregnant women, infants less than 1 year old, and individuals allergic to AZM, were given tetracycline ointments instead.

#### 3.1.2. Intervention

All studies took place in less-developed countries or disadvantaged communities. Two studies took place in Ethiopia [21, 22], three studies took place in Nepal [17, 19, 20], and two studies took place in Tanzania [18, 23]. One study targeted the aboriginal community in Northern Australia [16]. Four studies included untreated control arms [20–23].

There was considerable variation in the administration regimes used; azithromycin was administered once only at the beginning of five of the studies [16–19, 23], 3 monthly for 4 times in one study [22], annually for 3 times in one study [20], and biannually for 6 times in one study [21]. AZM dosage was mentioned in all but one study [23]. All specified the dosage as 20 mg/kg, while two studies also administered 1 gram for adults [19, 21].

#### 3.1.3. Sampling

There were largely two categories of sampling criteria for the eight included studies: random selection from a predefined age range [17, 19, 21–23] or all children within a specified age range [16, 18, 20]. Only one study specifically sampled children with trachoma [16]; other studies did not specify disease status.

### 3.2. Outcomes

#### 3.2.1. Primary: What Is the Effect of Azithromycin Treatment on Antibiotic Resistance among* S. pneumoniae* Isolates from the Nasopharynx?

The resistance status of* Streptococcus pneumoniae* to AZM was tested using different commercial tests: E-test© strips (AB Biodisk, Sweden and USA) [16, 18, 19, 22, 23] and broth dilution Sensititre© MIC plates (Trek Diagnostics Inc., USA) [20, 21]. One study mentioned the use of broth dilution MIC testing without specifying whether it was a commercial product [17].

Resistance status was determined by the MIC values as per test kit instructions; this was explicitly stated in six studies [16, 18–20, 22, 23]. The two remaining studies [17, 21] made reference to National Committee for Clinical Laboratory Standards, USA [24], and Clinical and Laboratory Standards Institute, USA [25], respectively.

The sampling process and subsequent transportation to laboratories were described in all studies. However, only four of the studies described the process of masking samples to laboratory workers [20–23].

Five studies [16–18, 22, 23] measured the baseline prevalence of resistance before AZM administration. The eight studies performed measurements at various time points after the antibiotic therapy, varying from once only at month 3 in Skalet's study [22] to month 6, year 1, and year 2 in Haug's study [21].

As previously mentioned, Haug 2010 and Skalet 2010, both based in Ethiopia, administered the antibiotic at higher frequencies, biannually and quarterly, respectively, than the rest of the included studies.

**Figure 2:**
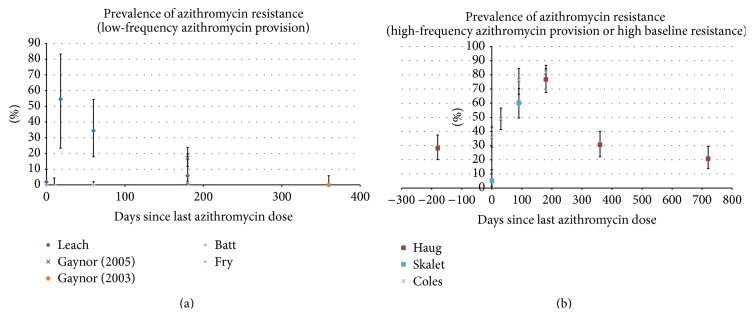
(a) Prevalence of azithromycin resistance amongst pneumococcus carriers plotted against time in studies with low-frequency azithromycin provision. The error bars show 95% confidence intervals about the proportions. (b) Prevalence of azithromycin resistance amongst pneumococcus carriers plotted against time in studies with high-frequency azithromycin provision or high baseline resistance. The error bars show 95% confidence intervals about the proportions.

All but two studies (Haug 2010 and Cole 2013) recorded low baseline prevalence of* S. pneumoniae* resistance to AZM (0% to 5.3% of isolates), which rose within three weeks following drug intake (0% to 54.6% of isolates) (Figure 2(a)). These studies showed the resistance figures dropping below 20% by 6 months and below 5% by 12 months.

Haug, Skalet, and Coles' studies showed much higher resistance rates at over 75% by 6 months [21–23] (Figure 2(b)). For Haug 2010 and Skalet 2010 studies, this was likely due to the more frequent AZM administration as discussed earlier, whilst, for Coles 2013 study, similarly high resistance values at baseline have also been demonstrated by the control group. These three studies appeared to display similar trends in the prevalence of resistance, with a prolonged peak of resistance at around 80% even at 6 months.

Five of the studies took baseline measurements [16–18, 22, 23] and four of the studies had a control arm [20–23]. However, due to the fundamental differences in the nature of these studies, both in terms of frequencies of AZM provision and varied baseline resistance, we did not combine the data for meta-analysis. It is of particular note that Coles 2013 demonstrated a high baseline resistance of 36% even without the prior dosing as in Haug 2010 study.

**Figure 3:**
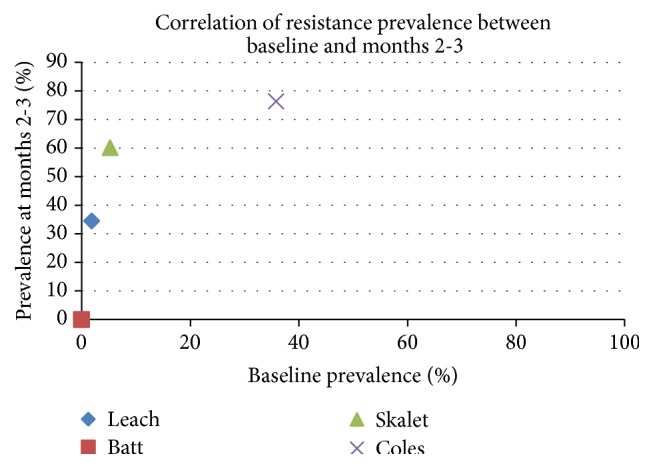
Correlation between prevalence of AZM resistance at baseline and months 2-3.

Examining the five studies where baseline resistance was recorded, the prevalence of resistance at months 2 to 3 appeared highly correlated with that of baseline, with a correlation coefficient of 0.759 (Figure 3).

#### 3.2.2. Secondary: What Is the Effect of Azithromycin Administration on the Prevalence of Nasopharyngeal Carriage of* S. pneumoniae*?

A variety of tests have been used to demonstrate the presence of the pathogen. These included* Streptococcus* selective media [19, 21, 22], morphology [16, 18], observation for *α*-haemolysis [18, 19], optochin susceptibility [16, 18, 19, 21–23], and bile solubility test [18, 19, 21, 22]. Two studies only described the use of media and sample freezing, but not the type of test used [17, 20].

**Figure 4:**
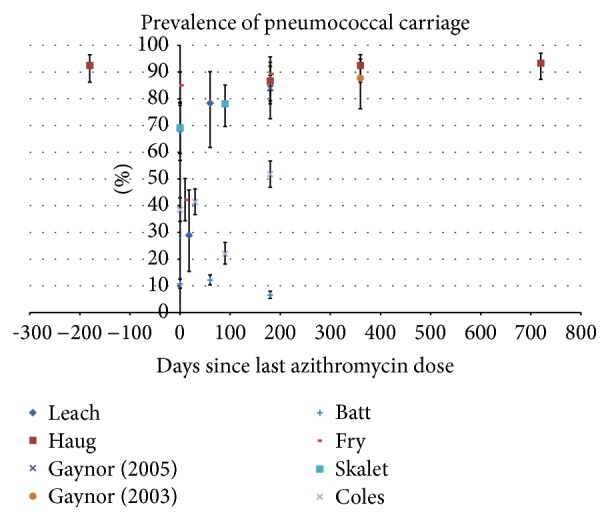
Prevalence of pneumococcal carriage, demonstrating the effect of azithromycin administration. The error bars show 95% confidence intervals about the proportions.

Prevalence of pneumococcal carriage in six of the eight studies ranged from 68% to 85% initially (Figure 4) and fell within days after AZM administration, returning to original values from 2 months onwards. Batt's and Coles' studies remained below 15% and 52% prevalence throughout the 6 months of their study periods, respectively.

### 3.3. Risk of Bias in Included Studies

#### 3.3.1. Selection Bias

Two of the studies [16, 19], instead of providing “blanket” AZM coverage to population, administered the antibiotic only to individuals suffering from active disease and their household contacts. The WHO simplified trachoma grading scale was used in one of these studies [19], but the number of assessors and the degree of their agreement were unknown. Leach did not report the criteria used at screening [16]. For studies with control groups, Skalet and Haug described the randomization process in treatment and control group allocations. This was absent in Gaynor's and Coles' studies; however. In Coles' study, the treated villages were chosen for their high trachoma prevalence (>10%), whilst the ineligible villages served as control group. Gaynor appeared to have included two randomly selected untreated villages only at the end of the study [20].

#### 3.3.2. Performance Bias

There was no masking of the study personnel, as the use of placebo was not mentioned in any of the included studies.

#### 3.3.3. Detection Bias

Assessments of nasopharyngeal streptococci carriage and antibiotic resistance are relatively easy to mask, as it is straightforward to anonymize laboratory samples. Three out of eight studies [20–22] reported masking of the outcome assessors (the laboratory workers) to the nasopharyngeal samples.

#### 3.3.4. Selective Reporting

Five out of eight studies reported sampling at baseline (i.e., before AZM was administered). Six studies reported outcomes at 6 months. The furthest follow-up point was 2 years. There was no suggestion from the published reports that the outcomes at any other time points were undisclosed due to selective reporting, although the lack of sampling at baseline made results interpretation more difficult due to the heterogeneity in the prevalence of resistance in different regions.

#### 3.3.5. Limitations of the Study

There were insufficient studies to rigorously assess reporting bias using funnel plots. We judged that there was no selective reporting of outcomes because the data was presented in full in all the included studies.

We included all studies irrespective of the language of publication; however, we cannot exclude the possibility that relevant studies published in languages other than English were not picked up in the initial search or that studies with negative findings were published in less accessible journals.

#### 3.3.6. Other Potential Sources of Bias

Background use of AZM or other erythromycins in the study regions could also render results less reliable, as the selection pressure would have been present prior to the studies. For the two studies in western Nepal [17, 20], around 3000 children were administered azithromycin as part of a clinical trial in 1998 [26]. For Coles' study, the reported use of (unspecified) drugs to treat suspected infections in the 30 days prior to study was over 65%. However, comparison with baseline or with a control arm in these studies mitigates against bias introduced by the use of antibiotics outside the study intervention. All other studies reported minimal background AZM use.

Azithromycin coverage also varies in different trials, as denoted in Table 1. For example, Batt administered AZM to all nonpregnant residents over 1 year old [18], while Coles administered the antibiotic only to children under 5 years old [23].

## 4. Discussion

*Streptococcus pneumoniae* is the most common causative pathogen for community-acquired pneumonia (CAP) [27]. Published guidelines from the Infectious Diseases Society of America (IDSA), European Society of Clinical Microbiology and Infectious Diseases (ESCMID), and British Thoracic Society (BTS) all reaffirm the role of macrolide as part of the initial empirical treatment for CAP in both outpatient and hospital settings [28–30]. This is particularly so in the US due to the higher prevalence of atypical organisms, whilst more emphasis is placed on penicillins in Europe and UK.

An European study on outpatient antibiotics use demonstrated a significant positive correlation between the volume of penicillin consumption in 19 countries and the prevalence of antibiotic resistance in* S. pneumoniae* [31]. This is of significance, because there may come a time for routine macrolide use when pneumococcal infections become commonly penicillin resistant.

A study that compared between annual and twice-yearly azithromycin regimes demonstrated that while a twice-yearly treatment can hasten the mean elimination time of ocular chlamydial infection by 7.5 months, the two groups showed no difference in disease prevalence from 18 months onwards [32].

Our systematic review faced some challenges as a result of limitations in the included studies. Three studies did not measure the baseline pneumococcal prevalence and antibiotic resistance. This made it difficult to prove that AZM provision resulted in any change. Four studies did not include a control arm and only 2 studies followed up their participants beyond 6 months, with Gaynor 2003 measuring 0% resistance to AZM among isolates at 1 year and Haug measuring 30.6%, which was similar to the baseline value of 28.2%. In terms of the strength of the included studies, all were cohort studies except Skalet 2010 and Haug 2010, which were randomized controlled trials.

A recent mathematical modeling study, based on the study data from Haug 2010, estimated that within 5 years of the last antibiotic dose there would be a 95% chance of macrolide resistance being eliminated by intraspecies competition [33]. However, it has also been suggested that sustained antibiotic use below a critical threshold may encourage the persistence of antimicrobial drug resistance [34].

Despite these limitations, it appears that in communities where baseline resistance to azithromycin in pneumococcus is low, mass AZM administration increased resistance only transiently, with the proportion of resistant cases gradually reducing as measurements were taken at further time points. A lack of long-lasting pneumococcal resistance may be somewhat reassuring for azithromycin-based trachoma eradication programs.

We however also noted two studies [21, 23] that demonstrated high baseline and subsequent antibiotic resistance in* Streptococcus*; one is the only study that implemented a high-intensity regimen for a prolonged period (twice a year for three years) [21], whilst the population examined in the other study appeared to have high background antibiotic use (65%–73% in control and treatment groups) [23]. These results from the field may give us an insight as to the potential adverse outcome when a critical threshold of antibiotic use in the region is exceeded. Our analysis, which showed a certain level of correlation of resistance prevalence between baseline and subsequent time point with a coefficient of 0.759, appears to be in agreement with this hypothesis.

Woolhouse and Farrar recently reiterated the importance of global efforts in combating antimicrobial resistance, with one of the possible solutions being the investigation of dosing regimens that can stall resistance development [35]. Health authorities in trachoma-affected regions should therefore be mindful of the selective pressure asserted by mass antibiotic use when implementing the SAFE strategy.

## Figures and Tables

**Table 1:** Characteristics of included studies.

Source	Country	Treatment group	Age range	Numbers treated	Control group	Treatment frequency	Measures baseline	Follow-up	Sample population	Test kit for resistance
Leach et al. 1997 [16]	Australia	Children with trachoma and their household contacts who were children	<15 years old	130	No	Single	Yes	2-3 weeks; 2 months; 6 months	All children <15 years old with trachoma	E-test© strips

Fry et al. 2002 [17]	Nepal	All children	1–10 years old	169	No	Single	Yes	10 days; 180 days	Randomly selected 1–10-year-old children	—

Batt et al. 2003 [18]	Tanzania	All nonpregnant individuals	>1 year old	4782	No	Single	Yes	2 months; 6 months	All children <7 year old	E-test© strips

Gaynor et al. 2003 [19]	Nepal	All children with clinically active trachoma and all household members of these children	1–10 years old	94	No	Single	No	1 year	Randomly selected 1–10-year-old children	E-test© strips

Gaynor et al. 2005 [20]	Nepal	All children	1–10 years old	194	Yes	Annual ×3	No	6 months	All children aged 1–7 years	Sensititre© MIC plates

Haug et al. 2010 [21]	Ethiopia	All nonpregnant individuals	>1 year old	Not known	Yes	Biannual ×6	No	6 months; 12 months; 24 months	Randomly selected 1–5-year-old children	Sensititre© MIC plates

Skalet et al. 2010 [22]	Ethiopia	All children	1–10 years old	3830	Yes	Quarterly ×4	Yes	3 months	Randomly selected <10-year-old children	E-test© strips

Coles et al. 2013 [23]	Tanzania	All children in MDA villages	<5 years old	467	Yes	Single	Yes	1 month; 3 months; 6 months	Randomly selected 2–5-year-old children	E-test© strips
